# Supplementary material for: Neurodevelopmental milestones and associated behaviours are similar among healthy children across diverse geographical locations
Source: Nat Commun. 2019 Jan 30;10:511. doi: 10.1038/s41467-018-07983-4 (PMC6353986; doi:10.1038/s41467-018-07983-4)
Supplement: Supplementary file 1 — Supplementary Information [file 41467_2018_7983_MOESM1_ESM.pdf]

Supplementary information

Supplementary Table 1. Baseline characteristics of the mothers of the 2 year old children in the INTERGROWTH-21st Project Neurodevelopment Assessment cohort.

	Brazil (n=199)	India (n=318)	Italy (n=311)	Kenya (n=311)	UK (n=168)
Maternal age (years)	28.4 (4.0)	26.8 (3.5)	28.7 (4.1)	28.6 (3.6)	30.1 (3.2)
Maternal height (cm)	162.9 (5.5)	158.5 (3.4)	163.5 (6.1)	162.2 (5.5)	166.1 (6.3)
Maternal weight (kg)	64.5 (8.7)	56.3 (8.4)	60.3 (8.1)	63.3 (9.1)	64.6 (8.8)
Paternal height (cm)	173.2 (6.2)	168.1 (5.6)	177.3 (8.9)	174.9 (5.7)	180.4 (6.8)
Maternal BMI (kg/m^2)	24.3 (2.9)	22.4 (3.1)	22.5 (2.7)	24.0 (3.1)	23.4 (2.8)
Gestational age at first ultrasound visit (weeks)	11.3 (1.3)	11.6 (1.6)	12.1 (0.8)	11.7 (1.5)	12.2 (1.1)
Years of formal education	13.6 (3.3)	16.1 (1.3)	13.8 (3.9)	15.5 (1.3)	16.3 (3.0)
Haemoglobin concentration <15 weeks' gestation (g/l)	12.5 (0.9)	11.7 (0.6)	12.9 (0.9)	13.0 (1.2)	12.7 (0.8)
Married or cohabiting	193 (97.0)	314 (98.7)	305 (98.1)	277 (89.1)	167 (99.4)
Nulliparous	147 (73.9)	229 (72.0)	195 (62.7)	198 (63.7)	111 (66.1)

Data are mean (SD) or n (%). BMI: body mass index.

Supplementary Table 2. Number of observations available for individual domains of the INTERGROWTH-21st Neurodevelopment Assessment, Cardiff tests and age of achievement of two WHO gross motor development milestones.

Domain	Brazil	India	Italy	Kenya	UK	TOTAL
**Cognitive**	199	317	310	309	159	1294
**Executive function-like**	199	317	310	310	161	1297
**Attentional problems**	199	318	311	311	141	1280
**Visual acuity**	198	315	305	288	161	1267
**Visual contrast sensitivity**	191	316	303	286	158	1254
**Fine motor**	199	317	310	309	159	1294
**Gross motor**	199	317	310	309	159	1294
**Receptive language**	199	317	310	309	157	1292
**Expressive language**	199	317	310	309	159	1294
**Positive behaviour**	199	317	310	310	161	1297
**Negative behaviour**	199	317	310	310	161	1297
**Total behaviour**	199	317	310	310	161	1297
**Emotional reactivity**	199	318	311	311	141	1280
**Positive affect**	199	318	311	311	141	1280
**Standing alone**	194	318	311	306	162	1291
**Walking alone**	194	318	309	309	164	1294
